# Baseline riparian forest and aquatic macrophyte vegetation dataset from the Danube right bank near Draž (Croatia), to support before-after monitoring of side-arm reconnection (2024-2025)

**DOI:** 10.3897/BDJ.14.e190669

**Published:** 2026-07-16

**Authors:** Dragana Vukov, Danijela Žunić, Nataša Prodanović, Vladimir Sabadoš

**Affiliations:** 1 Department of Biology and Ecology, Faculty of Sciences, University of Novi Sad, Novi Sad, Serbia Department of Biology and Ecology, Faculty of Sciences, University of Novi Sad Novi Sad Serbia; 2 Agricultural Extension Service “PSS Sombor”, Sombor, Serbia Agricultural Extension Service “PSS Sombor” Sombor Serbia

**Keywords:** aquatic macrophytes, baseline dataset, before-after monitoring, Danube River, ecological restoration, floodplain habitats, invasive species, riparian vegetation, side-arm reconnection, vegetation survey

## Abstract

**Background:**

Baseline biodiversity data collected prior to hydromorphological interventions are essential for understanding and evaluating ecological change following restoration or engineering measures. In large river floodplains, interventions such as side-arm reconnection are expected to alter hydrological regimes, habitat connectivity and disturbance patterns, with cascading effects on both riparian and aquatic plant communities. However, without well-documented pre-intervention datasets, post-restoration assessments often lack a reliable reference against which observed changes can be interpreted.

Along the right bank of the Danube River near the village of Draž (Croatia), planned hydro-technical measures aim to reconnect a cut-off side arm to the main channel. This reconnection is expected to modify local hydrological conditions and habitat structure in adjacent riparian forests and aquatic habitats, creating a clear need for baseline vegetation data collected prior to intervention.

**New information:**

This data paper presents a baseline dataset of riparian forest and aquatic vegetation collected along the right bank of the Danube River near the village of Draž (Croatia), prior to planned hydromorphological restoration measures involving the reconnection of a cut-off side-arm to the main channel. Riparian forest vegetation was surveyed in May 2024 using three 10 m × 10 m plots per site, with species cover estimated using the Domin cover-abundance scale and reported as plot means. Aquatic vegetation was surveyed in July 2025 using three 10 m belt transects per site, with species cover recorded in five ordinal cover classes and reported as transect means.

The dataset comprises 20 sampling events (10 forest and 10 aquatic), 251 taxon-by-event occurrence records and 331 measurements, including species cover or abundance, establishment status (native, introduced, invasive) and event-level environmental descriptors, such as canopy-cover classes, water physicochemistry, water-velocity class and CORINE Land-Cover categories. Taxonomic nomenclature was standardised according to Plants of the World Online (POWO 2026). The sampling design and data structure allow the dataset to serve as the “before” component of a before–after monitoring framework, supporting future assessments of vegetation responses to hydrological reconnection. The dataset provides a reference for restoration evaluation, invasion dynamics and comparative studies of riparian and aquatic plant communities in large river floodplains and constitutes the baseline vegetation component of the ecological monitoring programme established within the Horizon Europe project DaWetRest.

## Introduction

Baseline biodiversity data, collected prior to hydromorphological interventions, are essential for understanding and evaluating ecological change following restoration or engineering measures. In large river floodplains, interventions such as side-arm reconnection are expected to alter hydrological regimes, habitat connectivity and disturbance patterns, with cascading effects on both riparian and aquatic plant communities. Without well-documented pre-intervention datasets, post-restoration assessments often lack a reliable reference against which observed changes can be interpreted (Underwood 1994). Publishing such baseline datasets in standardised and reusable form ensures that future ecological assessments can distinguish restoration-induced changes from natural temporal variation and facilitates data reuse in comparative studies and long-term monitoring programmes (Chaven et al. 2017).

Along the Middle Danube floodplain near the village of Draž (Croatia), planned hydromorphological measures aim to reconnect a cut-off side arm to the main river channel. The study area forms part of the transboundary Middle Danube floodplain shared by Croatia and Serbia, where restoration initiatives increasingly aim to recover lateral connectivity and associated floodplain habitats. This floodplain belongs to one of the largest remaining riverine wetland systems in Europe and has been recognised for its exceptional biodiversity and ecological importance within the UNESCO Mura–Drava–Danube Biosphere Reserve. This reconnection is expected to modify local flow conditions, inundation dynamics and habitat structure, potentially reshaping vegetation composition in adjacent riparian forests and aquatic habitats (Tockner et al. 2009). River-floodplain systems are known to exhibit strong spatial and temporal heterogeneity and vegetation responses to altered hydrological connectivity are often gradual and context dependent (Tockner and Stanford 2002). Previous botanical investigations in the wider Croatian Danube floodplain, particularly in Kopački rit Nature Park, have documented high floristic richness, diverse wetland vegetation and the presence of numerous alien and invasive plant species (Rožac et al. 2018). The present dataset complements these regional floristic studies by providing a standardised, event-based baseline, specifically designed to support future restoration monitoring.

The dataset presented here documents the composition and cover of riparian forest and aquatic vegetation prior to the planned reconnection, using a standardised and repeatable sampling design. Riparian forests and aquatic habitats were surveyed separately, but within the same spatial framework, allowing the two vegetation compartments to be analysed independently or in combination. Species abundance was recorded using ordinal cover-abundance scales appropriate to each habitat type and complemented by event-level environmental descriptors, including canopy cover, water physicochemistry, water velocity class and land-cover context, thereby providing ecological information that extends beyond a simple species inventory. As the surveys were completed immediately before the planned restoration works, the dataset captures the baseline ecological condition against which subsequent post-restoration vegetation changes can be evaluated. In this context, the primary objective is not to characterise natural interannual variation, but to document the pre-intervention condition using a standardised sampling protocol that can be repeated during future monitoring.

Accordingly, the dataset was designed to represent the pre-intervention ("before") component of the ecological monitoring programme established within the Horizon Europe project DaWetRest. Although based on a single standardised pre-restoration survey, it provides the essential reference against which future post-restoration surveys can be evaluated. Together with subsequent monitoring, the dataset will support assessments of vegetation dynamics, habitat development, biological invasions and restoration effectiveness following side-arm reconnection. As both riparian forest and aquatic vegetation were surveyed using harmonised and repeatable sampling protocols and linked through a common sampling-event framework, the data also provide opportunities for comparative studies of vegetation composition and environmental relationships in large river floodplains. Unlike traditional floristic inventories, the present dataset was specifically designed to provide a standardised baseline for repeated vegetation surveys following hydromorphological restoration, thereby facilitating direct temporal comparisons within a long-term monitoring framework. Standardised pre-intervention datasets of this kind are an essential prerequisite for detecting ecological responses to restoration and evaluating restoration outcomes over time.

## General description

### Purpose

This dataset was collected within the Horizon Europe project DaWetRes (Danube Wetlands and flood plains Restoration through systematic, community engaged and sustainable innovative actions; project number: 101113015), funded under the call HORIZON-MISS-2022-OCEAN-01 (topic HORIZON-MISS-2022-OCEAN-01-02) as a Horizon Innovation Action (duration: 48 months) and managed by the European Climate, Infrastructure and Environment Executive Agency (CINEA). DaWesRes aims to restore ecological connectivity and ecosystem functioning in selected Danube floodplains through nature-based restoration measures. At the Croatian pilot-site near Draž, the principal restoration activity involves reconnecting a cut-off side arm to the main Danube channel in order to improve lateral hydrological connectivity. The vegetation surveys presented here constitute the baseline ("before") component of the ecological monitoring programme established to evaluate ecological responses to this intervention through future repeated surveys. The complete baseline dataset has been archived in the Zenodo repository to ensure long-term accessibility and reproducibility (https://doi.org/10.5281/zenodo.21023881).

## Sampling methods

### Study extent

Ten localities were sampled in riparian forest habitats and ten localities in aquatic habitats. Each sampling event corresponds to a site-level aggregation of three replicate sampling units. An overview of sampling events, site locations and key site-level characteristics is provided in Table 1.

### Sampling description

Riparian forest vegetation was surveyed on 20 May 2024 at ten localities. At each locality, three square plots (10 m × 10 m) were established within representative riparian forest stands so as to capture the dominant vegetation while avoiding recently disturbed microsites or habitat transitions. Plot localitions were selected to represent the prevailing vegetation structure at each locality and were positioned sufficiently apart to provide independent replicate samples. All vascular plant species occurring within each plot were recorded. Species cover was estimated using the Domin cover-abundance scale (0-9) and averaged across plots. Aquatic vegetation was surveyed on 30 July 2025 at the same ten localities. At each locality, three 10 m belt transects were established within the dominant aquatic vegetation generally parallel to the shoreline whenever local site morphology allowed, thereby ensuring comparable sampling amongst sites. Transects were positioned to represent the prevailing aquatic plant community while avoiding heavily disturbed or artificial microsites. Species cover was estimated using a five-level ordinal cover scale (1: > 1-5%; 2: > 5-25%; 3: > 25-50%; 4: > 50-75%; 5: > 75-100%) and averaged across the three transects.

Individuals located on plot or transect boundaries were included when the majority of their above-ground biomass occurred within the sampling unit.

All vegetation surveys were conducted by the same experienced botanist, using a standardised field protocol throughout the study. The use of a single observer minimised inter-observer variability in species identification and cover estimation, thereby improving the consistency and comparability of vegetation records across all sampling sites. Riparian forest and aquatic habitats were surveyed independently because of their contrasting vegetation structure and sampling requirements, while maintaining a common event-based sampling framework. The standardised methodology was selected to facilitate direct repetition during future post-restoration monitoring.

In addition to vegetation data, a set of site-level environmental descriptors was recorded for each sampling event. For forest sites, canopy cover was estimated using ordinal cover classes. For aquatic sites, water physicochemical parameters (pH, dissolved oxygen, conductivity and water temperature) were measured in the field and water velocity was classified into ordinal classes representing standing or slow-flowing conditions. Elevation and CORINE Land-Cover categories (CLC - European Union's Copernicus Land Monitoring Service (2018)), were recorded for all sampling events to provide additional spatial and landscape context.

### Quality control

Taxonomic identifications were carried out in the field by the same experienced botanist and standardised according to Plants of the World Online (POWO 2026), ensuring taxonomic consistency throughout the dataset. Event, occurrence and measurement identifiers were checked for consistency across the three relational tables prior to publication. Geographic coordinates, sampling dates and environmental variables were validated against original field records. Data consistency was further assessed by checking for duplicate records, missing identifiers and logical consistency amongst the linked event, occurrence and measurement tables and completeness of mandatory Darwin Core fields.

## Geographic coverage

### Description

Croatia, Osijek-Baranja County, vicinity of the village Draž; Danube right bank floodplain and adjacent aquatic habitats. Coordinates are included per sampling event in the dataset. The study area lies within the transboundary Middle Danube floodplain, a region characterised by high habitat heterogeneity resulting from interactions between the main river channel, side arms, floodplain forests and shallow aquatic habitats.

## Taxonomic coverage

### Description

Vascular plants recorded in riparian forest and aquatic habitats. The dataset includes 72 taxa in total, comprising 40 taxa recorded only in riparian forest habitats, 28 taxa recorded only in aquatic habitats and four taxa occurring in both habitat types (44 forest taxa; 32 aquatic taxa). Nomenclature was checked against POWO (2026). A complete list of recorded taxa, together with habitat association and establishment status is provided in Table 2.

## Temporal coverage

### Notes

Forest surveys: 20 May 2024. Aquatic surveys: 30 July 2025.

## Usage licence

### Usage licence

Open Data Commons Attribution License

## Data resources

### Data package title

Baseline riparian forest and aquatic vegetation dataset from the Danube right bank near Draž (Croatia) supporting before-after monitoring of side-arm reconnection (2024-2025). The dataset is deposited in the Zenodo repository and is publicly avalable at https://doi.org/10.5281/zenodo.21023881. The Zenodo record represents the authoritative, citable archive of the dataset, while CSV files provided as supplementary materials accompany this article for immediate access and inspection.

### Number of data sets

3

### Data set 1.

#### Data set name

Sampling events dataset

#### Data format

CSV

#### Character set

UTF-8

#### Description

Event-level dataset describing sampling events conducted in riparian forest and aquatic habitats along the right bank of the Danube River near Draž (Croatia). The dataset contains spatial coordinates, elevation, sampling dates, habitat classification and information on sampling protocols and sampling unit dimensions. The dataset is provided in Suppl. material 1.

**Data set 1.** 

Column label	Column description
eventID	Unique identifier of the sampling event.
eventID_raw	Original identifier of the sampling site used during field recording.
year	Year in which the sampling event was conducted.
habitat	Habitat type of the sampling site (riparian forest or aquatic habitat).
samplingProtocol	Description of the vegetation sampling method applied.
sampleSizeValue	Number of sampling units used within the event.
sampleSizeUnit	Unit describing the sampling unit (e.g. plots or transects).
plotSize_m2	Area of forest vegetation sampling plots in square metres.
transectLength_m	Length of aquatic vegetation belt transects in metres.
decimalLatitude	Latitude of the sampling site in decimal degrees.
decimalLongitude	Longitude of the sampling site in decimal degrees.
elevation_m	Elevation of the sampling site in metres above sea level.
eventDate	Date when the sampling event was conducted.

### Data set 2.

#### Data set name

Species occurrence dataset

#### Data format

CSV

#### Character set

UTF-8

#### Description

Occurrence-level dataset containing plant species records linked to sampling events. Each record includes the scientific name of the observed taxon, occurrence status, establishment status (native, introduced, invasive) and references used for taxonomic standardisation. The dataset is provided in Suppl. material 2.

**Data set 2.** 

Column label	Column description
occurrenceID	Unique identifier of each species occurrence record.
eventID	Identifier linking the occurrence record to the corresponding sampling event.
scientificName	Scientific name of the recorded plant taxon.
basisOfRecord	Type of record indicating that the occurrence was based on a human observation.
occurrenceStatus	Indicates whether the taxon was present in the sampling unit.
establishmentMeans	Establishment status of the taxon (native, introduced, invasive).
habitatType	Habitat category in which the occurrence was recorded.
identificationReferences	Reference used for taxonomic standardisation.

### Data set 3.

#### Data set name

Species abundance and environmental measurement dataset

#### Data format

CSV

#### Character set

UTF-8

#### Description

Measurement-level dataset containing species abundance data and environmental measurements linked to sampling events and species occurrences. Species cover values are recorded using the Domin scale for forest plots and ordinal cover classes for aquatic transects. Environmental variables include canopy-cover classes, water physicochemical parameters, water-velocity classes and CORINE Land-Cover categories. The dataset is provided in Suppl. material 3.

**Data set 3.** 

Column label	Column description
measurementID	Unique identifier of the measurement record.
occurrenceID	Identifier linking the measurement to the corresponding species occurrence record.
eventID	Identifier linking the measurement to the sampling event.
scientificName	Scientific name of the taxon for which the measurement was recorded.
habitatType	Habitat category in which the measurement was recorded.
measurementType	Type of measurement or environmental variable recorded.
measurementValue	Recorded value of the measurement.
measurementUnit	Unit associated with the measurement value.
measurementMethod	Method or scale used to obtain the measurement.

## Supplementary Material

1DA02EC3-BA4D-5A3A-AC16-91F9BB98D7AD 10.3897/BDJ.14.e190669.suppl1 Supplementary material 1 Event-level data (CSV) Data type sampling unit identifiers, geographic coordinates, elevation, sampling dates, habitat descriptions and sampling protocols Brief description Event-level table containing sampling unit identifiers, geographic coordinates, elevation, sampling dates, habitat descriptions and sampling protocols for riparian forest and aquatic sites. File: oo_1554993.csv https://binary.pensoft.net/file/1554993 Dragana Vukov, Danijela Žunić, Nataša Prodanović, Vladimir Sabadoš

E31561E7-3D78-52C6-83F9-FFD839146DD7 10.3897/BDJ.14.e190669.suppl2 Supplementary material 2 Occurrence-level data (CSV) Data type occurrences linked to sampling events, including establishment status (native, introduced, invasive) and taxonomic identification references Brief description Occurrence-level table containing taxon presence records linked to sampling events, including establishment status (native, introduced, invasive) and taxonomic identification references. File: oo_1554994.csv https://binary.pensoft.net/file/1554994 Dragana Vukov, Danijela Žunić, Nataša Prodanović, Vladimir Sabadoš

917422FD-7C0F-5201-ACA0-C0A2EF1EBB02 10.3897/BDJ.14.e190669.suppl3 Supplementary material 3 Measurement-level data Data type species cover/abundance data and event-level environmental variables Brief description Measurement-level table containing species cover/abundance data (Domin scale for forest plots; ordinal cover classes for aquatic transects) and event-level environmental variables (canopy-cover class, water physico-chemistry, water-velocity class and CORINE Land-Cover class). File: oo_1554995.csv https://binary.pensoft.net/file/1554995 Dragana Vukov, Danijela Žunić, Nataša Prodanović, Vladimir Sabadoš

## Figures and Tables

**Table 1.** Overview of sampling events and site-level charasteristics of riparian forest and aquatic habitats surveyed along the Danube right bank near Draž (Croatia). Canopy-cover classes: 2 = 5-25%; 3 = 25-50%; 4 = 50-75%. Water-velocity classes: 1 = no flow, stagnant; 2 = low flow. CLC European Union's Copernicus Land Monitoring Service (2018)- CORINE Land-Cover codes.

**eventID**	**habitatType**	**eventDate**	**latitude**	**longitude**	**elevation [m]**	**canopy cover**	**water velocity class**	**CLC**
forest_Lrnc1_1	forest	20-05-24	45.85311	18.80308	103	4	-	311
forest_Lrnc1_2	forest	20-05-24	45.85245	18.80394	100	4	-	311
forest_Lrnc2_1	forest	20-05-24	45.87516	18.81986	91	3	-	311
forest_Lrnc2_2	forest	20-05-24	45.87578	18.82291	93	3	-	311
forest_Lrnc2_3	forest	20-05-24	45.87535	18.82184	95	4	-	311
forest_Lrnc3_1	forest	20-05-24	45.88315	18.81778	88	4	-	311
forest_Lrnc3_2	forest	20-05-24	45.88253	18.81794	94	3	-	311
forest_Lrnc3_3	forest	20-05-24	45.88008	18.8191	105	4	-	311
forest_Topoljski	forest	20-05-24	45.84903	18.79994	93	2	-	324
forest_Sarkanj	forest	20-05-24	45.84716	18.80188	91	3	-	311
aquatic_Tplj1	aquatic	30-07-25	45.84734	18.79867	81	-	1	112
aquatic_Tplj2	aquatic	30-07-25	45.84722	18.79766	81	-	1	242
aquatic_Tplj3	aquatic	30-07-25	45.84726	18.79534	81	-	1	242
aquatic_Srknj1	aquatic	30-07-25	45.84878	18.80597	83	-	2	411
aquatic_Srknj2	aquatic	30-07-25	45.84869	18.80567	83	-	2	411
aquatic_Srknj3	aquatic	30-07-25	45.84784	18.80288	83	-	2	411
aquatic_Srknj4	aquatic	30-07-25	45.84744	18.80157	83	-	2	411
aquatic_Lrnc3	aquatic	30-07-25	45.8843	18.81829	87	-	1	411
aquatic_Lrnc2	aquatic	30-07-25	45.87553	18.82112	86	-	1	411
aquatic_Lrnc1	aquatic	30-07-25	45.85321	18.8046	84	-	1	411

**Table 2.** List of vascular taxa recorded in riparian forest and aquatic habitats prior to side-arm reconnection, with habitat association and establishment status. Nomenclature follows Plants of the World Online (POWO 2026).

**Scientific name**	**Habitat type**	**Establishment status**
*Acer negundo* L.	forest	invasive
*Agrostis stolonifera* L.	aquatic	native
*Alisma lanceolatum* With.	aquatic	native
*Alisma plantago-aquatica* L.	aquatic	native
*Alliaria petiolata* (M.Bieb.) Cavara & Grande	forest	native
*Ambrosia artemisiifolia* L.	aquatic	invasive
*Amorpha fruticosa* L.	forest	invasive
*Bidens tripartita* L.	aquatic	native
*Carex remota* L.	forest	native
*Carex* sp.	both	native
*Ceratophyllum* demersum L.	aquatic	native
*Cornus sanguinea* L.	forest	native
*Crategus* sp.	forest	native
*Cyperus michelianus* (L.) Delile	aquatic	native
*Echinochloa crus-galli* (L.) P.Beauv.	aquatic	introduced
*Epilobium angustifolium* L.	forest	native
*Euphorbia palustris* L.	aquatic	native
*Euphrosine xanthiifolia* (Nutt.) A.Gray	aquatic	introduced
*Frangula alnus* Mill.	forest	native
*Fraxinus angustifolia* Vahl	forest	native
*Fraxinus excelsior* L.	forest	native
*Galium aparine* L.	forest	native
*Galium palustre* L.	forest	native
*Geum urbanum* L.	forest	native
*Glechoma hederacea* L.	forest	native
*Humulus lupulus* L.	forest	native
*Hydrocharis morsus-ranae* L.	aquatic	native
*Iris pseudacorus* L.	both	native
*Juncus compressus* Jacq.	aquatic	native
*Lemna minor* L.	aquatic	native
*Leucojum vernum* L.	forest	native
*Lycopus europaeus* L.	aquatic	native
*Lysimachia nummularia* L.	forest	native
*Lysimachia vulgaris* L.	both	native
*Lythrum salicaria* L.	aquatic	native
*Morus alba* L.	forest	introduced
*Morus nigra* L.	forest	introduced
*Myosotis scorpioides* L.	forest	native
*Myriophyllum spicatum* L.	aquatic	native
*Najas marina* L.	aquatic	native
*Najas minor* All.	aquatic	native
*Panicum capillare* L.	aquatic	introduced
*Parietaria officinalis* L.	forest	native
*Persicaria amphibia* (L.) Gray	aquatic	native
*Persicaria hydropiper* (L.) Delabre	aquatic	native
*Persicaria mitis* (Schrank) Assenov	forest	native
*Phalaris arundinacea* L.	aquatic	native
*Phragmites australis* (Cav.) Trin. ex Steud.	aquatic	native
*Plantago major* L.	forest	native
*Populus alba* L.	forest	native
*Populus nigra* L.	forest	native
*Populus × canadensis* Moench	forest	introduced
*Prunus cerasifera* Ehrh.	forest	native
*Quercus robur* L.	forest	native
*Ranunculus repens* L.	forest	native
*Rhamnus cathartica* L.	forest	native
*Rorippa amphibia* (L.) Besser	both	native
*Rubus caesius* L.	forest	native
*Rumex sanguineus* L.	forest	native
*Salix alba* L.	forest	native
*Salvinia natans* (L.) All.	aquatic	native
*Spirodela polyrhiza* (L.) Schleid.	aquatic	native
*Stachys palustris* L.	forest	native
*Stellaria neglecta* (Lej.) Weihe	forest	native
*Stuckenia pectinata* (L.) Börner	aquatic	native
*Symphytum officinale* L.	forest	native
*Trapa natans* L.	aquatic	native
*Typha angustifolia* L.	aquatic	native
*Ulmus laevis* Pall.	forest	native
*Urtica dioica* L.	forest	native
*Vitis vinifera* L.	forest	native
